# DNA methylation age is accelerated in alcohol dependence

**DOI:** 10.1038/s41398-018-0233-4

**Published:** 2018-09-05

**Authors:** Allison D. Rosen, Keith D. Robertson, Ryan A. Hlady, Christine Muench, Jisoo Lee, Robert Philibert, Steve Horvath, Zachary A. Kaminsky, Falk W. Lohoff

**Affiliations:** 1 Section on Clinical Genomics and Experimental Therapeutics, National Institute on Alcohol Abuse and Alcoholism, National Institutes of Health, Bethesda, MD USA; 2 Department of Molecular Pharmacology and Experimental Therapeutics, Mayo Clinic, Rochester, MN USA; 3 Department of Psychiatry, University of Iowa, Iowa City, IA USA; 4 Department of Human Genetics, David Geffen School of Medicine, University of California Los Angeles, Los Angeles, CA USA; 5 Department of Biostatistics, Fielding School of Public Health, University of California Los Angeles, Los Angeles, CA USA; 6 Department of Psychiatry and Behavioral Sciences, Johns Hopkins University School of Medicine, Baltimore, MD USA; 7 Department of Mental Health, Johns Hopkins Bloomberg School of Public Health, Baltimore, MD USA

## Abstract

Alcohol dependence (ALC) is a chronic, relapsing disorder that increases the burden of chronic disease and significantly contributes to numerous premature deaths each year. Previous research suggests that chronic, heavy alcohol consumption is associated with differential DNA methylation patterns. In addition, DNA methylation levels at certain CpG sites have been correlated with age. We used an epigenetic clock to investigate the potential role of excessive alcohol consumption in epigenetic aging. We explored this question in five independent cohorts, including DNA methylation data derived from datasets from blood (*n* = 129, *n* = 329), liver (*n* = 92, *n* = 49), and postmortem prefrontal cortex (*n* = 46). One blood dataset and one liver tissue dataset of individuals with ALC exhibited positive age acceleration (*p* < 0.0001 and *p* = 0.0069, respectively), whereas the other blood and liver tissue datasets both exhibited trends of positive age acceleration that were not significant (*p* = 0.83 and *p* = 0.57, respectively). Prefrontal cortex tissue exhibited a trend of negative age acceleration (*p* = 0.19). These results suggest that excessive alcohol consumption may be associated with epigenetic aging in a tissue-specific manner and warrants further investigation using multiple tissue samples from the same individuals.

## Introduction

Alcohol dependence (ALC) is a chronic, relapsing disorder that significantly impacts personal and public health. ALC affects ~29.1% of individuals during their lifetime and is a leading cause of premature death in the United States^1^. In fact, between 2006 and 2010, chronic diseases caused by excessive alcohol use (such as hepatitis, hypertension, liver cancer, and stroke) resulted in 38,584 deaths and 864,351 years of potential life lost^2^.

Chronic alcohol use affects multiple tissues, including the brain and liver. Previous results found that ALC may be associated with accelerated decline in gray matter volume in the brain. In the prefrontal cortex (PFC), this decline could impact functioning, including cognitive abilities^3^. In addition, in a study using diffusion tensor imaging, middle-aged adults with ALC had reduced prefrontal white matter integrity and slower processing speed as compared to healthy controls^4^. Excessive alcohol use also has harmful effects on liver function that can lead to premature death due to alcoholic cirrhosis and liver cancer^5^.

Although the underlying pathophysiology is poorly understood, multiple genetic and epigenetic factors have been implicated in ALC and numerous chronic disease outcomes^6^. In fact, the field of epigenetics has shown that environmental exposures may lead to changes in gene expression that are not related to changes in the DNA sequence; these alterations occur via mechanisms such as DNA methylation and histone modification^7^. Specifically, DNA methylation profiles have been shown to differ between individuals who consume excessive amounts of alcohol and healthy individuals^8–10^.

The well-known strong associations that have been previously established between age and DNA methylation profiles suggest a new way to investigate aging on a molecular level^11–13^. Such research is increasingly important, as the United States Census Bureau estimates that the number of Americans over 65 years old will nearly double between 2012 and 2050^14^. A better understanding of molecular aging could allow for novel detection of aging-related morbidities and treatment methods, therefore improving overall health and well-being.

A growing interest in the development of biomarkers to study the relationship between aging and epigenetic profiles has led to the advent of epigenetic clocks. Horvath’s epigenetic clock, which is based on a weighted average of methylation levels at 353 dinucleotide cytosine phosphate guanine markers (CpGs), predicts DNA methylation age. Its high correlation with chronological age (years since birth), ability to predict all-cause mortality, and its validity across various tissue types (including blood, brain, and liver) make Horvath’s epigenetic clock a useful tool for the study of aging, as it has the potential to provide an accurate marker of biological age in clinical populations^12^.

Given the potential role of ALC in the aging process, it is important to gain a better understanding of this relationship on a molecular, epigenetic level. Although the relationship between ALC and aging has not been extensively studied using Horvath’s epigenetic clock, Quach et al.^15^ found a small negative correlation of age acceleration with moderate alcohol consumption in blood in an analysis of behavioral and lifestyle factors^15^. Similarly, an analysis using blood samples and a blood-specific epigenetic clock developed by Hannum revealed accelerated biological aging in light and heavy drinkers, and a deceleration in moderate drinkers^16^. Although these studies provide interesting preliminary findings, more specific studies focusing on alcohol consumption as the primary exposure are required to better understand the role of both moderate and heavy alcohol consumption in aging on the molecular level.

Developing an understanding of the role of ALC in the aging process is vital, as excessive alcohol use has been associated with numerous chronic disease outcomes and premature death, even following prolonged periods of sobriety^17^. In this study, Horvath’s epigenetic clock was used to investigate the cumulative role of ALC in molecular aging across tissue types including blood, brain, and liver. It was hypothesized that individuals with ALC would exhibit increased DNA methylation age acceleration as compared with healthy controls in all tissue types.

## Materials and Methods

DNA methylation age was calculated using Horvath’s epigenetic clock^12^. The epigenetic clock uses an algorithm based on 353 CpG sites to estimate DNA methylation age and is valid across different tissue types. The residual resulting from regressing DNA methylation age on chronological age was used as a metric for age acceleration. T-tests were used to compare age acceleration in individuals with ALC and healthy controls. Linear regression was used to control for sex, where age acceleration was the dependent variable, and ALC status and sex were the independent variables^13^. All analyses were conducted using R version 3.3.2 (R Foundation for Statistical Computing, Vienna, Austria) on the National Institutes of Health (NIH) Biowulf Linux cluster. Figures were generated using GraphPhad Prism 7 (GraphPad Software, Inc., La Jolla, CA). Analyses were performed using independent DNA methylation datasets derived from two datasets of blood tissue, two datasets of liver tissue, and one dataset of PFC tissue. The Diagnostic and Statistical Manual of Mental Disorders (DSM-IV) was used to diagnose ALC or alcohol abuse in all datasets with the exception of the liver dataset in which alcohol cirrhosis was used as a proxy for ALC. Detailed descriptions can be found below.

### Description of datasets

#### NIAAA blood sample

Fifty-nine participants with a diagnosis of ALC and 70 healthy volunteers were recruited to the National Institute on Alcohol Abuse and Alcoholism (NIAAA) at the National Institutes of Health (NIH), USA. The study was approved by the Institutional Review Board of the NIAAA and was in accordance with the Declaration of Helsinki. All individuals provided written informed consent and were compensated for their participation. The DSM-IV Structured Clinical Interview for Diagnosis was used to diagnose ALC^18^. All participants provided a blood sample. DNA methylation was measured using the Illumina Human Methylation 450 (HM450) beadchip microarrays (Illumina, Inc., San Diego, CA). The wateRmelon package in R was used to process the raw Illumina microarray data. Raw data were trimmed of probes failing quality assessment and a known list of 32,323 cross-reactive probes, followed by scale-based data correction for Illumina type I relative to type II probes. Methylated and unmethylated intensity values were then quantile normalized separately before the calculation of the *β*-value based on the following definition: *β*-value = (signal intensity of methylation-detection probe)/(signal intensity of methylation-detection probe + signal intensity of non-methylation-detection probe + 100). Values were then adjusted by taking the residuals of a linear model of *β*-values as a function of sodium bisulfite modification batch.

#### Grady Trauma Project sample (GSE72680)

The individuals in this study were part of an exploration of genetic and environmental factors that predict the response to stressful life events (such as interpersonal violence and psychosocial stress) in a predominantly African American, urban population of low socioeconomic status^19–22^. DNA methylation in blood samples was measured in 392 African American subjects using the Illumina HM450 beadchip microarray and DNA methylation *β*-values were downloaded from the Gene Expression Omnibus from GSE72680. Of these 392 individuals, 63 had missing alcohol phenotype data. This left 143 individuals with a diagnosis of ALC at some point in their lifetime and 186 controls. This dataset differs from others in that subjects were classified based on lifetime rather than current alcohol diagnosis.

#### University of Minnesota LTCDS liver cirrhosis sample

Forty-six liver samples from patients with alcoholic cirrhosis and 46 liver samples from healthy patients were obtained through the Liver Tissue Cell Distribution System (LTCDS, Minneapolis, Minnesota), which was funded by NIH Contract #HSN276200017C. DNA methylation *β*-values were measured at the NIAAA using the Illumina Infinium Methylation EPIC beadchip^9^.

#### Mayo liver cirrhosis sample (GSE 60753)

DNA methylation *β*-values measured using the Illumina HM450 beadchip microarray were downloaded from the Gene Expression Omnibus from GSE60753. Data consisted of 21 liver samples with alcoholic cirrhosis and 34 normal liver samples. The original study aimed to better understand the impact of environmental agents on DNA methylation and ultimately liver disease^23^. Two liver samples with alcoholic cirrhosis and four normal liver samples were eliminated due to missing data on chronological age. This resulted in the final analysis including 19 liver samples with alcoholic cirrhosis and 30 normal liver samples.

#### Postmortem PFC tissue sample (GSE49393)

DNA methylation *β*-values derived from postmortem PFC tissue were measured using Illumina HM450 beadchip microarrays from 46 PFC samples and were downloaded from the Gene Expression Omnibus from GSE49393^24^. The sample consisted of 23 subjects with DSM-IV diagnoses of ALC (*n* = 9) or alcohol abuse (*n* = 14) and 23 age-matched controls. Additional methodology can be found elsewhere^24^. The New South Wales Tissue Resource Centre at the University of Sydney provided fresh-frozen sections of Brodmann area 9 (mainly the dorsolateral PFC of the brain) postmortem brain tissues. Ethics approval was obtained from the Sydney Local Health Network and the University of Sydney.

## Results

Horvath’s epigenetic clock was used to study epigenetic aging in five datasets. Sample sizes and demographic information for each sample can be found in Table 1. Mean age ranged from 30 to 57 years old, with individuals with ALC being slightly older than controls in most samples. All but the Grady Trauma Project (GSE72680) sample were predominantly male.

**Table 1:** Sample and demographic information

	**Chip**	**Sample size**		**Chronological sge** mean (SD)		**Male sex** count (%)		**Citation**
		**Case**	**Control**	**Case**	**Control**	**Case**	**Control**	
**NIAAA** *Blood*	450k	59	70	39.98 (10.02)	30.40 (8.38)	43 (72.88%)	36 (51.43%)	This paper
**GSE72680 (Grady Trauma Project)** *Blood*	450k	143	186	43.70 (10.67)	40.08 (14.29)	65 (45.45%)	30 (16.13%)	GSE72680
**UMN LTCDS** *Liver*	EPIC	46	46	54.00 (8.52)	54.15 (8.53)	41 (89.13%)	41 (89.13%)	This paper
**GSE60753 (Mayo)** *Liver*	450k	19	30	57.52 (9.64)	55.45 (17.82)	16 (84.21%)	14 (46.67%)	GSE60753
**GSE49393** *PFC*	450k	23	23	57.01 (9.26)	56.04 (9.40)	16 (69.57%)	16 (69.57%)	GSE72680

**Fig. 1:**
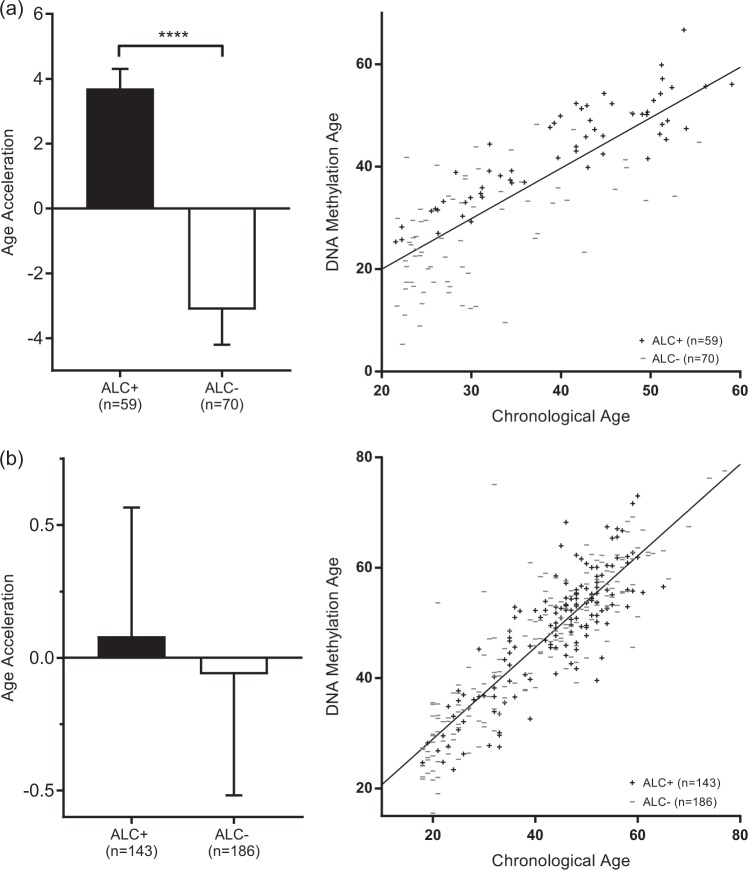
Age acceleration in blood. **(a)** NIAAA blood sample. **(b)** Grady Trauma Project (GSE72680) blood sample. Bar plots show average age acceleration and 1 SE, as reported in Table 2. Scatter plots show chronological age vs. DNA methylation age and a line in which DNA methylation age was regressed on chronological age. Points lying above the line exhibit negative age acceleration and points lying below the line exhibit positive age acceleration. In both samples, age acceleration was positive in cases and negative in controls. Average age acceleration differed significantly (*p* < 0.0001) between cases and controls in the NIAAA sample, but not in the Grady Trauma Project sample

In the NIAAA blood sample, average age acceleration was positive in individuals with ALC and negative in healthy controls. This difference was highly significant (*p* <0.0001). The Grady Trauma project showed a trend in the same direction; however, age acceleration did not significantly differ between those with a diagnosis of ALC in their lifetime compared to those without (see Table 2 and Fig. 1 for additional information)

**Table 2:** Average age acceleration (calculated using the residual resulting from regressing DNA methylation age on chronological age) and SE for samples

	**Average age Acceleration**		**SE**		***P*** **-value**
	**Case**	**Control**	**Case**	**Control**	
**NIAAA** *Blood*	3.703	− 3.121	0.600	1.082	**<** **0.0001**
**GSE72680** *Blood*	0.081	− 0.063	0.484	0.456	0.8302
**UMN LTCDS** *Liver*	1.488	− 1.488	0.792	0.689	**0.0069**
**GSE60753** *Liver*	0.864	− 0.547	2.390	1.074	0.5649
**GSE49393** *PFC*	− 0.577	0.577	0.574	0.659	0.1932

*Note.* Boldface indicates significance. Average age acceleration differed significantly in the NIAAA blood sample and the UMN liver sample.

**Fig. 2:**
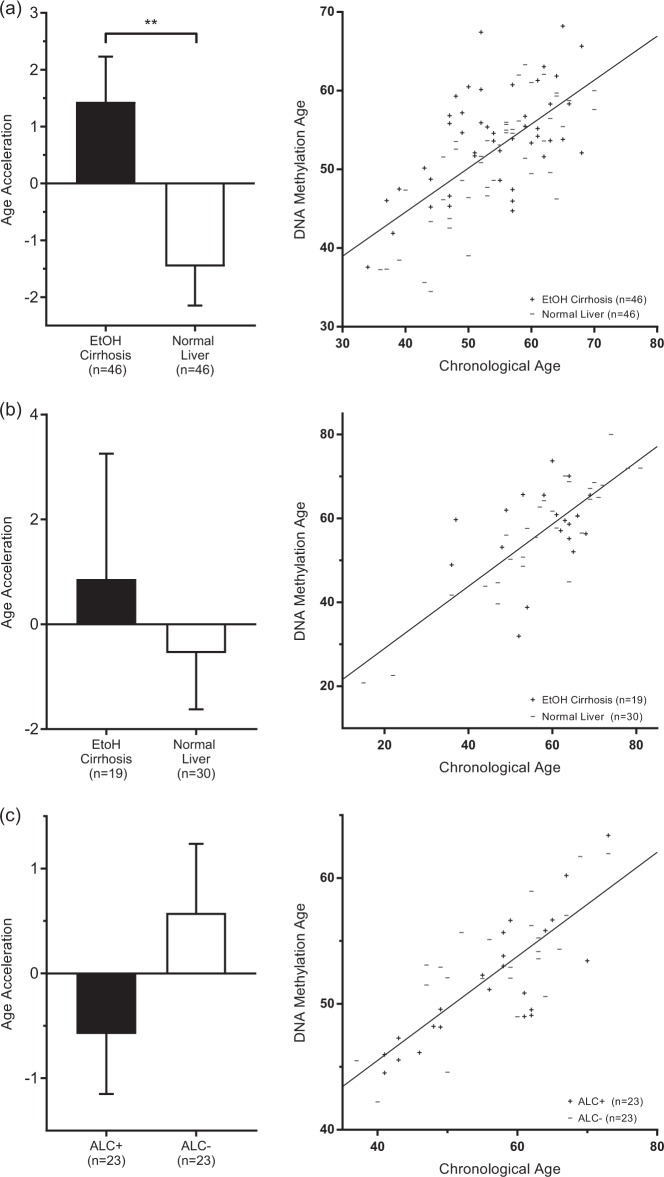
Age acceleration in liver and prefrontal cortex. **(a)** UMN liver sample. **(b)** Mayo Clinic liver sample (GSE60753). **(c)** Australian Brain Bank (GSE49393) prefrontal cortex sample. Bar plots show average age acceleration and 1 SE, as reported in Table 2. Scatter plots show chronological age vs. DNA methylation age and a line in which DNA methylation age was regressed on chronological age. Points lying above the line exhibit negative age acceleration and points lying below the line exhibit positive age acceleration. In liver samples, age acceleration was positive in cases and negative in controls. Average age acceleration differed significantly (*p* = 0.0069) between cases and controls in the UMN sample, but not in the Mayo Clinic Sample. In prefrontal cortex, age acceleration was negative in cases and positive in controls. Average age acceleration did not differ significantly between cases and controls

Similar to the blood samples, The University of Minnesota LTCDS sample showed positive age acceleration in cirrhotic liver tissue and negative age acceleration in healthy liver tissue. These results must be interpreted with some caution; the epigenetic clock is optimized for the Illumina 27k and 450k chips, but this data was generated using the Illumina EPIC chip, which contains > 90% (but not all) of the markers included on the others and thus required the epigenetic clock algorithm to use mean imputation. The same trend was observed in the Mayo liver sample. The difference between groups was significant (*p* = 0.0069) in the University of Minnesota LTCDS sample, but not in the Mayo liver sample. See Table 2 and Fig. 2 for additional information.

The dataset of PFC tissue showed negative age acceleration in those with a diagnosis of ALC/alcohol abuse and positive age acceleration in healthy controls, but the averages did not differ statistically significantly. As a result of a small amount of missing data due to errors in measurement of DNA methylation *β*-values on the Illumina chip, these findings should be taken with some caution. See Table 2 and Fig. 2 for additional information.

Linear regression models showed no significant relationship between sex and age acceleration in each of the five samples. In addition, ALC status was significantly associated with age acceleration in the NIAAA sample and the University of Minnesota LTCDS sample when controlling for sex. See Table 3 for additional information. Lastly, exploratory analyses conducted separately for males and females in each sample did not reveal any novel statistically significant differences with regard to age acceleration (data not shown).

**Table 3:** Results of linear regression models where age acceleration is the dependent variable and ALC status (ALC + or ALC −) and sex (male or female) are the independent variables

		**ALC Status**		**Sex**	
	***y*** **-Intercept**	***β***	***p***	***β***	***p***
**NIAAA** *Blood*	3.7904	− 6.8497	**1.15** **×** **10**^**-6**^	− 0.1201	.9303
**GSE72680** *Blood*	0.7646	− 0.1452	0.8380	− 0.9863	0.2040
**UMN LTCDS** *Liver*	0.3277	− 3.0439	**0.0063**	1.3564	0.4202
**GSE60753** *Liver*	3.8730	− 1.4430	0.6110	− 4.5440	0.1170
**GSE49393** *PFC*	− 0.4443	1.1549	0.1980	− 0.1914	0.8430

*Note.* Boldface indicates significance. The reference categories are ALC − and male, respectively. In line with the results of the *t*-tests in Table 2 and Figs. 1 and 2, alcohol use was significantly associated with age acceleration in the NIAAA sample and UMN sample. Sex was not significant in any of the five samples, suggesting that these findings hold after controlling for sex

## Discussion

In this study, we explored for the first time epigenetic aging within multiple datasets and tissue types of individuals with ALC and controls. We observed positive age acceleration in one blood dataset and one liver tissue dataset, but not in brain tissue. These results are particularly interesting, given the known negative effect of prolonged excessive alcohol consumption on these parts of the body^1^.

The positive age acceleration trend observed in each of the cirrhotic liver samples was consistent with our hypothesis. However, we expected to see a greater magnitude of positive age acceleration, as both datasets consisted of samples from individuals with alcoholic cirrhosis, a severe end-stage disease caused by particularly heavy alcohol consumption. Analyses conducted in such tissue must be interpreted with caution, as hepatocytes found in cirrhotic liver tissue differ significantly from healthy hepatocytes and DNA methylation might vary^5^. In fact, previous research shows that the process of alcoholic cirrhosis may directly influence DNA methylation. In addition, cell composition differences in cirrhotic liver versus normal liver might account for changes in DNA methylation age, as not only the hepatocytes, but also cells linked to fibrosis and inflammation can be abnormal in alcohol cirrhosis^25^. One way to address this limitation would be the use of Andres Housman’s reference-free method epigenetic clock, which accounts for cell type-specific DNA methylation effects and different cell proportions^26^. It remains unknown to what degree progressive alcoholic liver damage correlates with increased epigenetic aging, as longitudinal liver biopsy samples from individuals across the life span are not available to our knowledge. Although both liver datasets show age acceleration in alcohol cirrhosis, future larger studies are needed that in particular take into account various demographic covariates as well as cell-specific analyses to confirm this finding.

ALC is strongly associated with degeneration of brain tissue as well as premature cognitive decline^3,4,27,28^. Thus, we expected to find positive age acceleration in PFC tissue, rather than the negative age acceleration that we observed. However, there may be an explanation for these findings; of our sample of 23 participants, 14 (61%) were diagnosed with DSM-IV alcohol abuse and only nine (39%) were diagnosed with ALC. Alcohol abuse is a milder phenotype than ALC and is thus associated with significantly lower levels of consumption. In fact, it is possible that this sample is more representative of a population that consumes alcohol at moderate levels, which some research suggests may be associated with healthy aging, reduced risk of coronary heart disease, and reduced risk of cognitive decline and Alzheimer’s disease^29–34^. Future larger studies should confirm the effects of alcohol on epigenetic age in PFC tissue and might also explore additional brain regions, as results in our PFC sample may not represent those in other regions.

Studying epigenetic aging in the blood is important, as such results may be combined with those from other tissues to eventually permit the use of DNA methylation signatures in blood as a useful biomarker of aging and to assess age acceleraton across the life span. The difference in results between the NIAAA sample and Grady Trauma project sample may be explained by the multi-year difference in age both within and between the two samples^13^. In addition, the NIAAA sample was characterized by a current ALC diagnosis, as well as current desire for inpatient treatment, while the Grady Trauma Project was characterized by a diagnosis of ALC anytime in one’s lifetime. Thus, the NIAAA sample may represent a more severe phenotype than the Grady Trauma project sample. In fact, previous research indicates that differences in genome-wide DNA methylation in individuals with ALC and controls may be diminished following periods of abstinence^35^. In addition, false reporting is common when patients are asked to self-report alcohol use, and may significantly influence the make-up of the case and control groups. This highlights the importance of developing useful biomarkers to measure alcohol use^36^. Finally, the Grady Trauma project was primarily a study of childhood trauma, which may explain the small difference between cases and controls. We did not control for this possible covariate but it might have an effect on our results as a previous epigenetic clock analysis using this data found that cumulative lifetime stress was associated with accelerated aging^37^.

Further analyses would benefit from increased statistical power. Many of our datasets had sample sizes ranging from 25 to 50 individuals per group, which may partially explain or influence these findings. In addition, clinical heterogeneity remains a concern for studies of individuals with substance use disorders and the comparison of multiple datasets can be problematic. For example, the average chronological age in the NIAAA blood sample was approximately 15 years younger than that of the postmortem PFC sample and two liver cirrhosis samples. In addition, ALC has been shown to be associated with a multitude of comorbidities including cardiovascular disease, liver disease, smoking, and other substance use disorders, as well as psychiatric disorders, such as major depressive disorder and bipolar I disorder^1^. Previous studies suggest that comorbidities and other lifestyle factors, in combination with the medication used to treat them, are likely to influence epigenetic aging and should be controlled for in future analyses^13,15^.

Given that the epigenetic clock is valid across different tissue types, collecting blood, liver, and postmortem brain tissue samples from the same individuals would not only remedy this problem of clinical heterogeneity, but also allow us to take full advantage of the capabilities of the epigenetic clock. Furthermore, it would be beneficial to acquire data on race and ethnicity and specific drinking patterns/abstinence in these samples, as well as to create a more equal balance of males and females, as recent research has shown that both race and sex may play a role in the epigenetic aging process^13,38^. Finally, future studies of epigenetic aging in ALC might consider analysis of longitudinal data, especially after a period of treatment or sobriety, to investigate the stability of age acceleration in different tissue types in this population.

In conclusion, the present study provided evidence that epigenetic aging differs in blood and liver tissue of individuals with ALC compared to healthy volunteers. Although there were limitations in this study, these results warrant further investigation into the role of chronic, heavy alcohol consumption in the aging process across various tissue types.
